# Urban Retail Food Environments: Relative Availability and Prominence of Exhibition of Healthy vs. Unhealthy Foods at Supermarkets in Buenos Aires, Argentina

**DOI:** 10.3390/ijerph18030944

**Published:** 2021-01-22

**Authors:** Natalia Elorriaga, Daniela L. Moyano, María V. López, Ana S. Cavallo, Laura Gutierrez, Camila B. Panaggio, Vilma Irazola

**Affiliations:** 1 Institute for Clinical Effectiveness and Health Policy (IECS), Department of Research on Chronic Diseases, Ciudad Autónoma de Buenos Aires C1414CPV, Argentina; dmoyano@iecs.org.ar (D.L.M.); mvlopez@iecs.org.ar (M.V.L.); ascavallo@iecs.org.ar (A.S.C.); lgutierrez@iecs.org.ar (L.G.); virazola@iecs.org.ar (V.I.); 2 National Scientific and Technical Research Council (CONICET), Center for Research on Epidemiology and Public Health (CIESP-IECS), Ciudad Autónoma de Buenos Aires C1414CPV, Argentina; 3 Escuela de Nutrición, Universidad de Buenos Aires (UBA), Ciudad Autónoma de Buenos Aires C1121ABG, Argentina; 4 Departamento de Salud, Universidad Nacional de La Matanza (UNLaM), San Justo B1754JEC, Argentina; camilabpanaggio@gmail.com

**Keywords:** healthy food retail, retail food environment, supermarket, food availability, Buenos Aires, INFORMAS, socio-economic factors

## Abstract

There is growing evidence that the food environment can influence diets. The present study aimed to assess the relative availability and prominence of healthy foods (HF) versus unhealthy products (UP) in supermarkets in Buenos Aires, Argentina and to explore differences by retail characteristics and neighborhood income level. We conducted store audits in 32 randomly selected food retails. Food availability (presence/absence, ratio of cumulative linear shelf length for HF vs. UP) and prominence inside the store (location visibility) were measured based on the International Network for Food and Obesity/NCDs Research, Monitoring and Action Support (INFORMAS) protocol. On average, for every 1 m of shelf length for UP, there was about 25 cm of shelf length for HF (HF/UP ratio: 0.255, SD 0.130). UP were more frequently available in high-prominence store areas (31/32 retails) than HF (9/32 retails). Shelf length ratio differed across commercial chains (*p* = 0.0268), but not by store size or type. Retails in the lower-income neighborhoods had a lower HF/UP ratio than those in the higher-income neighborhoods (*p* = 0.0329). Availability of the selected HF was overcome largely by the UP, particularly in high prominence areas, and in neighborhoods with lower income level, which may pose an opportunity for public health interventions.

## 1. Introduction

Obesity rates are increasing in Latin America and globally [1,2]. In Argentina, 68% of adults and 41% of children and adolescents aged 5–18 are now overweight or obese [3]. Moreover, the obesity prevalence in Argentina shows a clear inverse association with income and educational level [3,4]. Like other countries in the region, the dietary pattern of the population in Argentina has shifted in recent years as a result of cultural changes and modifications in food accessibility, such as an increase in the consumption of ultra-processed products and a decrease in the consumption of healthy fresh and minimally processed foods such as fruit, vegetables and pulses [1,5,6,7]. In 2013, the average expenditure per household in ultra-processed products represented 28% of the total expenditure on foods and beverages nationwide, being higher in locations with a higher level of urbanization [8]. The role of unhealthy food environments in shaping transitioning diets in low- and middle-income countries is increasingly gaining policy attention [9]. Food environments, defined as the collective, economic, policy and social surroundings, opportunities and conditions that influence people’s food and beverage choices and nutrition status [10], are considered one of the main drivers of the obesity pandemic [11]. An important component of the nutrition environments is the in-store retail food environment or consumer nutrition environment, which refers to what consumers encounter at the location where they buy foods, including nutrition quality, product placement, promotions, price range and nutritional information of the available products in the stores [12]. In this study, we focused on the first two characteristics, availability of healthy and unhealthy products—according to the Argentine Dietary Guidelines [13,14] and their strategic placement inside the store, in retail food environments in Buenos Aires (CABA), the capital and most populated city of Argentina.

In Argentina, supermarkets are an important part of the community nutrition environment, including both non-discount and discount stores, which are a particular type of supermarket that typically offer products that are priced lower than in other supermarkets [15,16,17,18]. In addition to large supermarket chains, there are independent (non-chain) supermarkets, about 80% of which are owned by Chinese independent grocers and are called “Chinese Supermarkets” [19,20]. The community nutrition environment in the City of Buenos Aires also includes other retailers such as specialized small grocery stores (e.g., ‘green-grocers’), itinerant food fairs, kiosks and restaurants [19,21]. It has been estimated that supermarket chains with the greatest number of stores and the largest sales space sell 58% of the total food and beverages in Argentina [16]. Moreover, during the last decade, these large chains have opened smaller branches [19]. As a result, between 2011 and 2017, the number of supermarkets in the city doubled [22].

Little is known about how healthy the consumer environment in supermarkets in Buenos Aires is, and whether there are differences between supermarkets taking into account characteristics such as size (e.g., the small supermarkets vs. the larger ones), type (e.g., discount vs. non-discount supermarkets) and chains, or among neighborhoods of different income levels. To our knowledge, with a few exceptions [23,24,25], little research has been published about the topic in Latin America, since most studies have been conducted in developed countries. Some of their findings are that stores of different size, type and chain may differ in the healthiness of the retail food environment [18,26,27,28]. It has been shown that although larger supermarkets offer more shelf space to fruits and vegetables than other types of stores, they may also devote more shelf space to unhealthy snacks [26], and that the relative availability may vary from urban to non-metropolitan stores [27]. Discount supermarkets may have little availability of some products [18,29]. Additionally, a large variation in the relative availability of healthy vs. unhealthy products within and across supermarket chains has been reported, suggesting that there may be room for improvement in this regard [28]. Finally, recent research suggests that in socio-economically disadvantaged neighborhoods, both the exposure to energy-dense snack foods and soft drinks in supermarkets may be greater, and the relative availability of healthy vs. unhealthy products may be lower than in wealthier areas [28,30].

The retail food environment is a potential setting to implement interventions to promote healthy diets [31]. Growing evidence suggests an association between the consumer food environment and dietary outcomes; however, some mixed results have been shown from systematic reviews, partly due to the variety and complexity of methods used to assess the availability of foods within stores [32,33,34,35]. The International Network for Food and Obesity/NCDs Research, Monitoring and Action Support (INFORMAS) has developed methods and indicators to measure and benchmark food environments, including retail food environments, among countries. In the present study, we used an adapted version of the Module Food Retail-Food Availability in Supermarkets developed by INFORMAS [36].

This study aimed to assess the relative availability and prominence of exhibition of healthy versus unhealthy foods in supermarkets in the City of Buenos Aires, Argentina. A secondary aim was to explore differences in the availability and prominence by outlet characteristic, including the supermarket size (small vs. medium/large), type (discount vs. non-discount stores) and chain, as well as the neighborhood income level.

## 2. Materials and Methods

### 2.1. Design and Sample

This is a cross-sectional study that was conducted in the City of Buenos Aires, Argentina. The city, with a population of 3,075,646 inhabitants, has an area of 203 km^2^ and is administratively organized in 15 neighborhoods located in three zones (north, central and south). Eligible supermarkets were those with at least two checkouts. The location of the stores was obtained from company websites and other online directories. Supermarkets were classified by location (neighborhood 1 to 15) and size (small-sized and medium/large-sized). Supermarket outlet classification by size is detailed in Appendix A.

The sample frame included 579 small-sized and 244 medium/large-sized supermarkets. Based on data from previous studies, we calculated a minimum sample size of 30 stores to be able to estimate the mean shelf length ratio with a margin of error equal to 10% of the mean or less [37]. We randomly selected 32 stores by means of the random number function in Microsoft Excel. Randomization was stratified by neighborhood and size of the supermarket to ensure that at least one small and one medium/large supermarket was selected in each neighborhood.

Data for the present study were collected from April to June 2019.

### 2.2. Ethical Appoval

The protocol of the study was approved by the Institutional Review Board of the Hospital Italiano de Buenos Aires (IRB00010193, Ethical approval code: 3846, 6 September 2018).

### 2.3. Definition of Indicators and Variables

We calculated the following indicators: (a) the presence of selected healthy and unhealthy food categories in the stores, (b) the cumulative shelf length in meters (m) devoted to these food categories and (c) the relative availability of healthy and unhealthy foods (healthy foods/unhealthy foods shelf length ratio) estimated as the sum of the shelf lengths occupied by healthy products divided by the sum of the shelf lengths occupied by unhealthy products. All the indicators were estimated in general for each supermarket and stratified by areas of different prominence inside the stores.

The INFORMAS protocol to assess the food availability in supermarkets recommends the use of an indicator of the relative availability of food and beverages [36,37]. In this study, the indicator was adapted to be used in Argentina by including other healthy items besides fruits and vegetables. The selection of the foods and beverages has been based on a supplementary study (more details are included in Appendix B). The adapted indicator includes shelf length measurements taken for five types of healthy foods displayed and four categories of unhealthy products. Selected healthy foods were: fruits and vegetables—both fresh and frozen—whole grains without added sugars, pulses, unsalted nuts and water. Selected categories of unhealthy products were sweet biscuits/cookies, confectionery (including chocolate), crisps and sugar-sweetened beverages—including sodas, flavored water and artificial juices.

Prominence assessment of in-store locations was based on the GroPromo tool [38], which takes into account the customer exposure to the area where the products are placed. In-store areas were classified into high prominence (e.g., checkout side and checkout end and aisle endcaps that face the checkouts or the center of the store), medium prominence (e.g., endcaps that face the back or the perimeter of the store, aisles and islands) and low prominence (e.g., edges).

Supermarket size was classified into two categories: small-sized—including the smaller branches of chain supermarkets, with a sales space surface between 200 m^2^ and 400 m^2^—and medium/large-sized, including independent or chain stores with a sales space larger than 400 m^2^ of sales space. Supermarket type was categorized as either a discount or non-discount supermarket, as detailed in Appendix A. Supermarket chains of the outlets included in the sample were assigned a letter (A to D). The neighborhood income level was defined based on the mean level of household income per capita in Buenos Aires in 2019 [39]. In this work, neighborhoods were classified into two categories using the median value (AR$33,454).

### 2.4. Data Collection

Data for the present study were collected by three observers: two dietitians and a nurse (D.L.M., A.S.C. and C.B.P.). Training consisted of two sessions of two-hour instruction, followed by four hours of practice under the supervision of the lead researcher (N.E.) or the supervisor (D.L.M.) in two retail stores. The shelf length ratio measures were conducted across supermarkets following the standard protocol developed by INFORMAS [28,36]. Briefly, linear shelf length of the selected food categories was measured in meters by two researchers using an inextensible measuring tape, either along the shelf or along the floor in front of the shelf. The number of shelves of equal length on which the target food was displayed was also recorded and multiplied by the linear shelf length to obtain the cumulative shelf length for each food category. For shelving units that did not have a physical shelf (e.g., units with hanging confectionery), a row of hanging products was counted as a single shelf. Displays that contained multiple rows of different products (e.g., dividers between frozen food) were counted as multiple ‘shelves’ in the same way. Measurement of islands or freestanding bins was performed by measuring the exposed sides from which customers could pick the products. For round freestanding bins, the diameter was measured and the circumference calculated using 2πr.

To calculate the inter-rater reliability, a second researcher assessed three supermarkets on the same day as the first one. Intra-class correlation coefficient for cumulative shelf length for selected categories and by prominence area was calculated to assess inter-rater reliability. Measures of shelf length within the food categories show very good inter-rater reliability: intra-class correlation coefficient was 0.917 for cumulative shelf length for the selected food categories, and 0.933 for the shelf length by prominence area.

### 2.5. Data Management and Analysis

Data were collected on paper forms and then entered into the study database, which was designed using the REDCap electronic data capture tools hosted at IECS [40,41].

We calculated the proportion of supermarkets in which each food category was available and its 95% confidence intervals (95% CI). Since some categories of foods and beverages were available in 100% of the supermarkets, in those cases a one-sided 97.5% CI was calculated. The Chi square and Fisher´s exact tests were conducted to compare the proportion of supermarkets with availability of the food categories according to the characteristics of the supermarkets.

Continuous variables, such as the shelf length assigned to each category of healthy and unhealthy foods and the shelf length ratio of healthy to unhealthy foods, were described by mean, standard deviation (SD), median and interquartile range (IQR). The Kruskal–Wallis test was conducted to compare the shelf length for each food category, the cumulative shelf length of healthy and unhealthy foods and the ratio of shelf length of healthy to unhealthy foods, by prominence of location inside the store. To evaluate the differences in the ratio of shelf length devoted to healthy vs. unhealthy foods across supermarkets of different size, type, chain and neighborhood income level, the Wilcoxon rank-sum and Kruskal–Wallis tests were performed. A *p*-value < 0.05 was considered statistically significant. Data were analyzed using Stata/SE 12.0 for Windows (Stata Corp LP, College Station, TX, USA, 2011).

## 3. Results

### 3.1. Characteristics of the Sample

**Figure 1:**
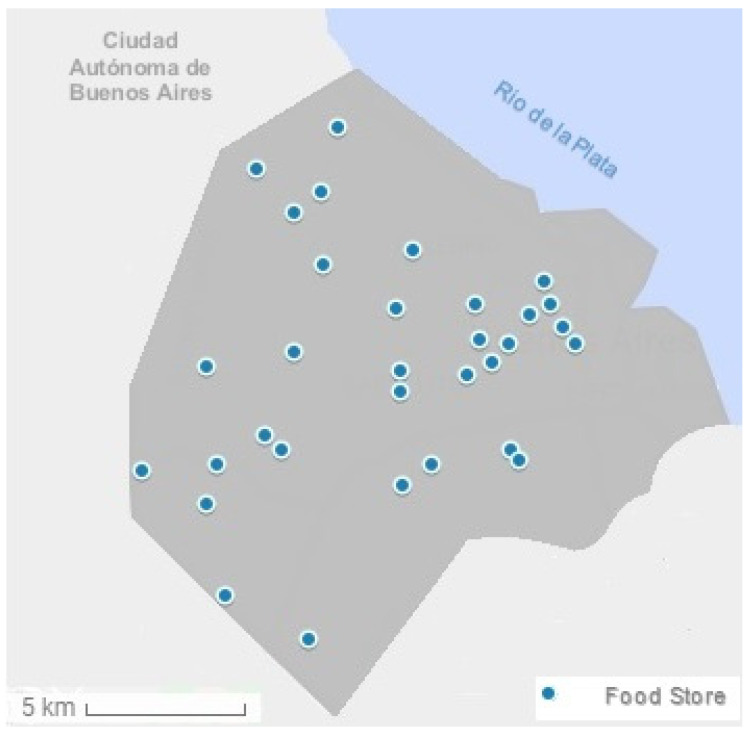
Location of the retail food stores included in the study.

Characteristics and localization of the stores included in the study are summarized in Table 1 and Figure 1.

### 3.2. Availability of Healthy and Unhealthy Items by Prominence of Location Inside the Store

Table 2 shows the presence of the selected food items in the 32 food retails by prominence of location inside the store. Each of the four categories of unhealthy products was available in all the audited stores; however, availability of healthy items varied across stores, with beans having the lowest availability (available in 17 supermarkets) to water having the highest availability (available in 31 supermarkets). Availability of food products differed by prominence area inside the store. In locations of high prominence, such as checkouts and endcaps facing the checkouts, there was at least one unhealthy item in most of the retails (31/32), while only 9 out of the 32 stores had at least one of the healthy products available.

### 3.3. Shelf Length Assigned to Food and Beverages Items and Relative Shelf Length of Healthy and Unhealthy Items by Prominence of Location Inside the Store

**Figure 2:**
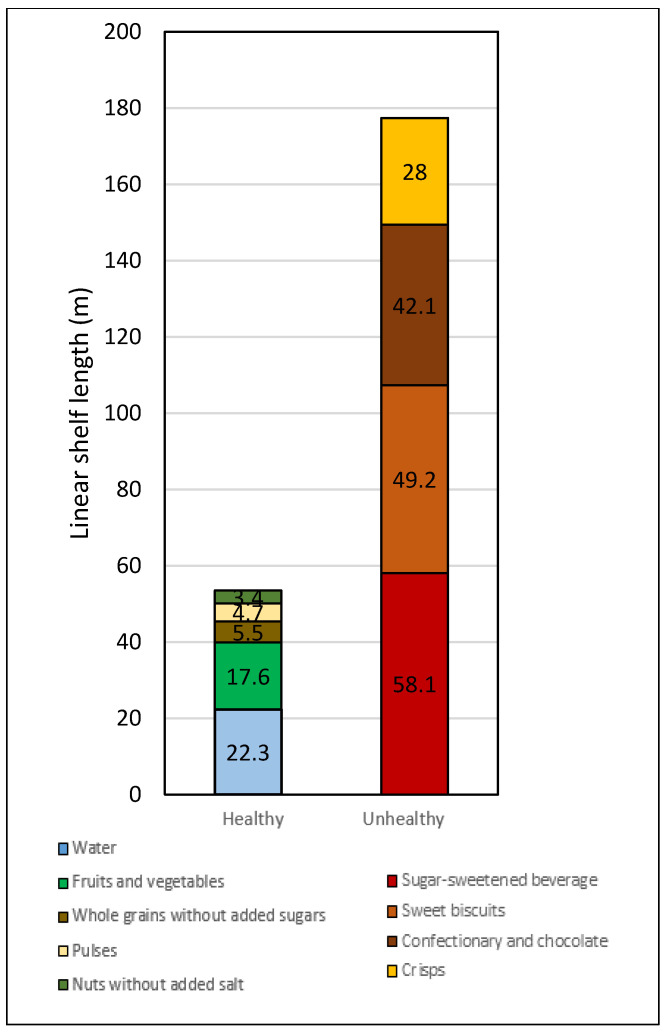
Average cumulative linear shelf of healthy and unhealthy items in supermarkets in the City of Buenos Aires (*n* = 32).

Table 3 and Figure 2 show the average linear shelf length devoted to healthy and unhealthy foods and their ratio. Overall, the average shelf length was 53.4 m for healthy foods and 177.4 m for unhealthy products. This means that for every 1 m of shelf length for unhealthy foods, there was a shelf length of about 25 cm for healthy foods.

**Figure 3:**
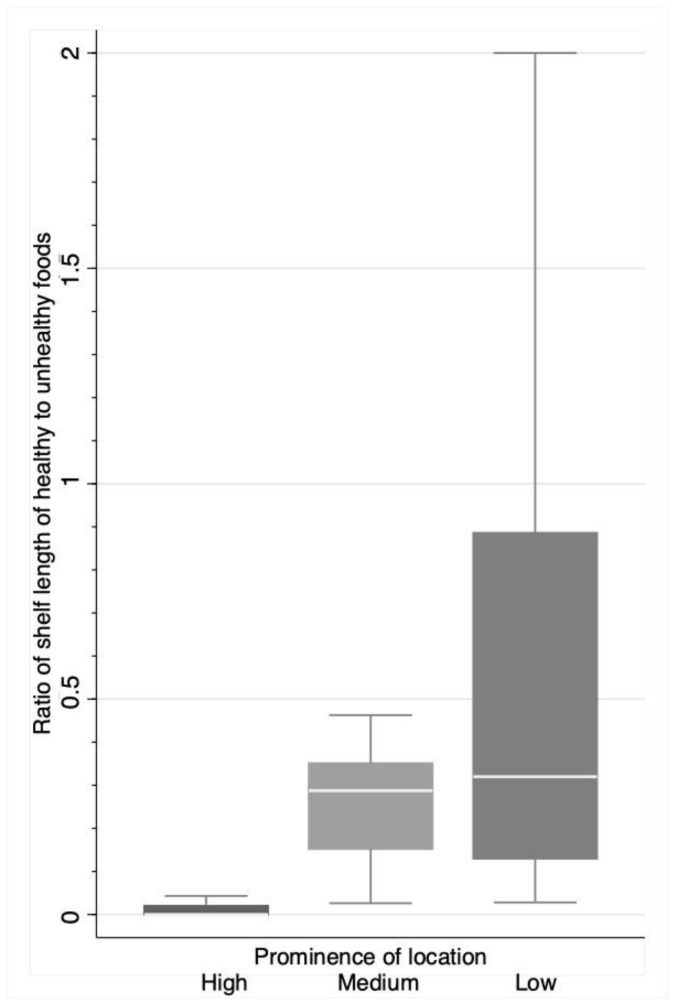
Median ratio of shelf length of healthy to unhealthy foods by prominence location in supermarkets in the City of Buenos Aires (*n* = 32). Box squares represent 25th and 75th percentiles. Kruskal–Wallis test (*p* = 0.003). High prominence areas: checkout side and checkout end and aisle endcaps that face the checkouts or the center of the store. Medium prominence areas: endcaps that face the back or the perimeter of the store, aisles and islands. Low prominence areas: edges.

An analysis of the same indicators by prominence of location is shown in Table 3 and Figure 3. There were differences in the shelf length assigned to most of the food items in areas of different prominence inside the store, with the exception of pulses and confectionary. The ratio of shelf length of healthy to unhealthy foods differed according to the prominence area inside the stores (*p* = 0.003). The ratio of shelf length of healthy to unhealthy foods differed according to the prominence area inside the stores (Table 3 and Figure 3). In high prominence areas, space devoted to unhealthy items, measured in shelf length, largely overcame the space devoted to healthy foods (19.10 m vs. 0.31 m), with a mean healthy/unhealthy ratio of 0.013. In places of medium and low prominence inside the supermarkets, the space assigned to unhealthy items was also greater than the space assigned to healthy foods, but differences were smaller (mean ratio 0.417 and 0.846).

### 3.4. Availability and Relative Linear Shelf Length of Healthy and Unhealthy Items by Supermarket Characteristics and Neighborhood Income Level

As was previously explained, the selected unhealthy products were available in all the stores. Healthy items were found with similar frequency in both medium/large and small supermarkets as well as in those stores located in neighborhoods of different income levels (data not shown). The presence of the majority of healthy items was also similar at discount and non-discount supermarkets; however, the availability of pulses was almost null in discount supermarkets (1/13), being higher in non-discount supermarkets (16/19) (*p* < 0.0001).

Table 4 shows the average ratio of cumulative linear shelf length for healthy versus unhealthy foods by supermarket characteristics and neighborhood income level. The ratio was similar through categories of stores size and type. However, there were some variations between commercial chains, with a median ratio ranging from 0.168 to 0.396 (*p* = 0.0268). A lower shelf length ratio of healthy vs. unhealthy foods was found in neighborhoods with lower income in comparison with higher-income neighborhoods (*p* = 0.0329).

## 4. Discussion

To our knowledge, this is the first study to assess relative food availability, overall and by prominence, in the retail food environment in an urban setting in Argentina. The study shows that all the assessed categories of unhealthy products were present in all the audited stores; instead, the availability of the assessed healthy foods was variable and depended on the food categories. Our results also indicate that in the supermarkets of the city, the shelf length devoted to the four categories of unhealthy food products was on average four times as large as the shelf length assigned to the five healthy food categories, suggesting an obesogenic retail food environment. The study conducted in New Zealand (NZ) by Vandevijvere et al. [28] used the same methodology obtaining comparable results; however, the stores in Buenos Aires seems to have a lower shelf length ratio (CABA: 0.25 SD 0.13 vs. NZ: 0.42, SD 0.13), meaning a worse relative of healthy vs. unhealthy products. Our findings on the availability of foods by prominence of location were as expected [28], that is, the ratio of shelf length of healthy to unhealthy foods was lowest in high prominence areas in the stores. Studies conducted in other countries have also shown that the strategic placement of discretionary products, such as soft drinks and unhealthy snacks, was commonly observed in supermarkets [27,42].

Our study also explored differences in food availability across food outlets of different sizes, types and commercial chains. Differences in relative availability of fruits and vegetables vs. unhealthy snack foods were previously described between stores of different sizes, reporting healthier ratios in larger supermarkets [26]. In this work, small stores showed a similar shelf length ratio to larger stores, but in both categories, the ratio was lower than the one previously described for supermarkets [26]. Because in our study we collapsed super- and hypermarkets into the same category, further studies including a higher sample size separating those categories may have to be conducted to assess these differences. Otherwise, it has been reported that discount stores may have a limited assortment of products and little availability of fresh produce [18,29]. Our study suggests that the overall shelf length ratio was similar in traditional and discount stores in Buenos Aires. However, one of the healthy food categories, the pulses, showed almost no availability in discount supermarkets. While national dietary guidelines recommend increasing the consumption of pulses—Argentina produces and exports pulses, and they are relatively affordable—the national consumption is low [14]. Discount supermarkets are usually used more frequently by people in the lower socio-economic levels than those in the higher ones [43]. Thus, ensuring the availability of these healthy and relative affordable foods in these kinds of outlets would help to improve accessibility in this subgroup of the population [9]. Beyond pulses, since we have only evaluated nine categories of food and beverages, further research is needed to assess whether other types of foods are available in discount supermarkets in Buenos Aires in similar quantities as those in non-discount supermarkets. Lastly, large variations in the availability of healthy and unhealthy products between and within retail chains have been reported by previous studies [28,44]. Our results are in accordance with those studies, and show variation in terms of the shelf length ratio among stores, suggesting that there is room for improvement.

Many studies have reported differences in the consumer nutrition environment across neighborhoods of different socio-economic levels [24,25,28,30,32]. In agreement, our findings also suggest that the stores in neighborhoods with lower income levels offer a lower relative availability of healthy vs. unhealthy products. These inequities are in accordance with those reported about national food consumption; in comparison to people in the higher income level, those in the lowest levels of income reported less frequent consumption of fruits, vegetables and other healthy foods, and highly frequent consumption of SSBs and other discretionary ultra-processed products such as salted snacks and crisps, pastry products and confectionary [3].

In summary, many of the problems identified as nutrition health priorities in Argentina could be related to the retail food environment, as has been shown in the present study: overweight and obesity, overconsumption of refined sugars, sodium and sugar-sweetened beverages, increased consumption of sweets, confectionery and overall discretionary foods and low consumption of fiber, water, fruit, vegetables and pulses [14]. As has been exposed, there are inequities in diets and obesity prevalence according to the socio-economic level of the population. We have studied food availability and prominence dimensions of the consumer nutrition environment in the City of Buenos Aires, suggesting an obesogenic environment. Monitoring of these indicators will help to assess changes in the retail food environment over time. Recent interventions targeting other dimensions of the food environment have been successfully introduced in Argentina and other Latin American countries. Examples of these interventions are food reformulation [45,46], taxes on sugary drinks [47], front labeling of packaged foods [48,49,50] and improving school food environments [51], and most of them have the potential to influence the quality of the products available in food outlets and their promotions. Interventions directly targeting the retail food environment have been less studied in Latin America; however, there is evidence indicating that the amount of shelf space allocated to foods influences consumer purchases and have been related with body mass index [33,52,53,54]. A recent review indicated that most of the interventions targeting in-store products, promotions, price and placement showed at least some positive effect on consumer purchases and/or dietary intake [31]. However, there have been some mixed or null results, which could be explained partially by the inaccuracy of methods to assess outcomes [31,55,56]. In Argentina, regulations limiting the availability of certain unhealthy ultra-processed products at and near the checkouts of stores have recently been approved in three provinces, Neuquén (Act 3224/2019), Río Negro (Act 5383/2019) and La Pampa (Act 3248/2020). In addition, other similar law projects have been introduced in other provinces as well as in the City of Buenos Aires. Further studies should evaluate their implementation and assess their effects.

One strength of this study is that the stores included were randomly selected, including retailers from all the neighborhoods of the CABA. Another is that, with a minimal adjustment, we used an audit tool previously validated by INFORMAS, showing very good test-retest reliability in our study. However, there are some limitations of this study. It is important to acknowledge that the foods assessed may not reflect the entire range of foods available to consumers; however, the use of simple indicators measuring a selection of healthy and unhealthy food categories increases the feasibility of conducting this kind of study and is similar to several studies conducted in the field [23,24,26,27,30,37]. In addition, we included only small-sized chain supermarkets and super- and hypermarkets; hence, our results do not consider the full consumer food environment, and further research to adapt and test the audit tool to smaller specialized stores and food fairs should be warranted. Finally, we have explored some differences across stores and neighborhood income level; to further study these differences, other designs and a larger sample size would be necessary.

## 5. Conclusions

In conclusion, our results indicate that, on average, for every 1 m of shelf length for unhealthy foods, there was 25 cm of shelf length for healthy foods, with lower shelf length ratio in high prominence locations in supermarkets in Buenos Aires, suggesting an obesogenic retail food environment. Our study also suggests that the ratio of shelf length of healthy to unhealthy foods varied across commercial chains, without differences by size of store. Lower average shelf ratios were found in neighborhoods of lower income level. Discount supermarkets show a similar ratio to non-discount supermarkets, but the availability of some healthy foods may be lower in the former. Our findings can help inform policy makers and the civil society to take action in improving food environments for consumers in Argentina through regulations, public campaigns, and other policy strategies to reduce obesity- and diet-related non-communicable disease risk factors. Future research needs to examine changes over time, differences in other types of stores, and the effect of interventions over purchasing and dietary behavior.

## Figures and Tables

**Table 1:** Characteristics and geographical location of stores included in the study.

Characteristics of the Supermarkets	*n*	%
**Type of food retail**		
Non-discount store	19	59.3
Discount store	13	40.6
**Outlet size ^1^**		
Small	16	50.0
Medium/large	16	50.0
**Geographical Zone**		
North	7	21.8
Central	16	50.0
South	9	28.1
**Neighborhood income level**		
≤median income per capita	17	53.1
>median income per capita	15	46.9

^1^ Median area was 322 m^2^ for small-sized supermarkets and 1386 m^2^ for medium/large-sized supermarkets.

**Table 2:** Presence of selected food items in supermarkets by areas of different prominence inside the store. City of Buenos Aires, Argentina (*n* = 32).

Item	Any Place in the Store	Prominence ^1^		
		High	Medium	Low
	*n*	*n*	*n*	*n*
	% (95% CI)	% (95% CI)	% (95% CI)	% (95% CI)
Water	31	6	26	26
	96.9 (83.8–99.9)	18.8 (7.2–36.4)	81.3 (63.6–92.8)	81.3 (63.6–92.8)
Fruits and vegetables	29	0	20	21
	90.6 (75.0–98.0)	0 (0.0–10.9) *	62.5 (43.7–78.9)	65.6 (46.8–81.4)
Pulses	17	0	14	4
	53.1 (34.7–70.9)	0 (0.0–10.9) *	43.8 (26.4–62.3)	12.5 (3.5–29.9)
Whole grains without added sugars	30	1	24	17
	93.8 (79.2–99.2)	3.1 (0.1–16.2)	75.0 (56.6–88.5)	53.1 (34.7–70.9)
Nuts without added salt	26	2	19	6
	81.2 (63.6–92.8)	6.3 (0.8–20.8)	59.4 (40.6–763)	18.8 (7.2–36.4)
Any healthy product	32	9	32	32
	100.0 (89.1–100.0) *	28.1 (13.7–46.7)	100.0 (89.1–100.0) *	100.0 (89.1–100.0) *
Sugar-sweetened beverages	32	17	32	30
	100.0 (89.1–100.0) *	53.1 (34.7–70.9)	100.0 (89.1–100.0) *	93.8 (79.2–99.2)
Crisps	32	14	27	15
	100.0 (89.1–100.0) *	43.8 (26.4–62.3)	84.4 (67.2–94.7)	46.9 (29.1–65.3)
Sweet biscuits	32	7	29	14
	100.0 (89.1–100.0) *	21.9 (9.3–39.9)	90.6 (75.0–98.0)	43.8 (26.4–62.3)
Confectionary and chocolate	32	29	23	10
	100.0 (89.1–100.0) *	90.6 (75.0–98.0)	71.9 (53.6–86.3)	31.3 (16.1–50.0)
Any unhealthy product	32	31	32	31
	100.0 (89.1–100.0) *	96.9 (83.8–99.9)	100.0 (89.1–100.0) *	96.9 (83.8–99.9)

95% CI, 95% confidence interval * One-sided, 97.5% confidence interval; ^1^ High prominence areas: checkout side and checkout end and aisle endcaps facing the checkouts or the center of the store. Medium prominence areas: endcaps facing the back or the perimeter of the store, aisles and islands. Low prominence areas: edges.

**Table 3:** Average linear shelf length (m) of healthy and unhealthy products and ratio by prominence areas inside the food retails in the City of Buenos Aires, Argentina (*n* = 32).

Item	Any Place in the Store		Prominence ^1^						*p*-Value ^3^
			High		Medium		Low		
	Mean (SD)	Median (IQR)	Mean (SD)	Median (IQR)	Mean (SD)	Median (IQR)	Mean (SD)	Median (IQR)	
Water	22.3 (38.2)	13.0 (6.9–19.8)	0.2 (0.6)	0.0 (0.0–0.0)	16.9 (30.1)	9.8 (2.0–17.8)	5.2 (9.2)	2.4 (0.6–4.6)	0.001
Fruits and vegetables	17.6 (25.9)	10.0 (2.1–19.5)	0.0	0.0 (0.0–0.0)	8.9 (13.7)	3.12 (0.0–10.4)	8.7 (14.8)	1.2 (0.0–9.3)	0.003
Pulses	4.7 (16.8)	0.6 (0.0–2.3)	0.0	0.0 (0.0–0.0)	1.9 (3.9)	0.0 (0.0–2.2)	2.7 (14.9)	0 (0.0–0.0)	0.452
Whole grains without added sugars	5.5 (10.9)	2.8 (1.5–3.9)	0.0 (21.2)	0.0 (0.0–0.0)	3.6 (8.8)	1.0 (0.1–2.9)	1.9 (2.8)	0.3 (0.0–3.3)	0.032
Nuts without added salt	3.4 (7.6)	1.3 (0.6–2.3)	0.1 (0.3)	0.0 (0.0–0.0)	2.9 (7.5)	0.8 (0.0–2.1)	0.4 (0.9)	0.0 (0.0–0.0)	0.021
Healthy foods (total length)	53.4 (85.4)	25.9 (19.5–38.4)	0.3 (0.7)	0.0 (0.0–0.4)	34.2 (55.0)	17.6 (12.0–29.5)	19.0 (31.4)	6.9 (2.9–18.8)	0.002
Sugar-sweetened beverage	58.1 (82.1)	33.6 (25.9–53.4)	2.1 (3.1)	0.6 (0.0–2.7)	42.3 (68.7)	24.4 (12.5–40.3)	13.8 (15.8)	10.4 (5.5–15.4)	<0.001
Crisps	28.0 (18.3)	22.0 (15.6–31.7)	1.3 (3.0)	0.0 (0.0–1.0)	19.3 (19.9)	15.6 (3.8–25.2)	7.4 (11.3)	0.0 (0.0–13.0)	<0.001
Sweet biscuits	49.2 (55.6)	37.9 (28.3–49.7)	0.9 (2.4)	0.0 (0.0–0.0)	41.4 (55.2)	32.0 (23.6–42.2)	7.0 (15.2)	0.0 (0.0–4.5)	<0.001
Confectionary and chocolate	42.1 (57.1)	24.4 (15.7–44.6)	14.9 (17.8)	8.5 (4.3–19.0)	19.3 (34.1)	7.35 (0.0–20.0)	8.0 (19.8)	0.0 (0.0–7.2)	0.1948
Unhealthy products (total lenght)	177.5 (20.3)	115.0 (90.5–178.2)	19.1 (21.9)	10.8 (5.8–22.5)	122.3 (166.1)	77.5 (54.0–116.4)	36.0 (36.5)	27.8 (11.3–51.4)	<0.001
Ratio healthy/unhealthy products	0.255 (0.130)	0.232 (0.169–0.283)	0.013 (0.026)	0.0 (0.0–0.014)	0.417 (0.753)	0.271 (0.150–0.350)	0.846 (1.366) ^2^	0.320 (0.130–0.886)	0.003

IQR, Interquartile range; SD, Standard Deviation. ^1^ High prominence areas: checkout side and checkout end and aisle endcaps that face the checkouts or the center of the store. Medium prominence areas: endcaps that face the back or the perimeter of the store, aisles and islands. Low prominence areas: edges. ^2^ For low prominence, there was one zero value for the cumulative linear shelf length of unhealthy foods. ^3^ *p*-value for the Kruskal–Wallis test.

**Table 4:** Ratio of cumulative linear shelf length for healthy versus unhealthy foods by zone and supermarket characteristics.

Subgroups	*n*	Mean (SD)	Median (IQR)	*p*-Value
Overall	32	0.255 (0.130)	0.232 (0.169–0.284)	
**Location by income level**				0.0329
Lower-income neighborhoods	17	0.208 (0.086)	0.206 (0.163–0.246)	
Higher-income neighborhoods	15	0.309 (0.152)	0.273 (0.208–0.359)	
**Outlet size**				0.9622
Small	16	0.246 (0.111)	0.226 (0.188–0.276)	
Medium/Large	16	0.264 (0.150)	0.244 (0.151–0.326)	
**Food retail type**				0.2577
Non-discount supermarket	19	0.246 (0.143)	0.206 (0.141–0.306)	
Discount supermarket	13	0.268 (0.113)	0.263 (0.225–0.277)	
**Supermarket Chain ^1^**				0.0268
A	8	0.199 (0.068)	0.188 (0.152–0.248)	
B	6	0.379 (0.177)	0.396 (0.246–0.553)	
C	13	0.268 (0.113)	0.263 (0.225–0.277)	
D	5	0.161 (0.068)	0.168 (0.134–0.197)	

IQR, Interquartile range; SD, Standard Deviation. ^1^ Different letters represent different chains.

## Data Availability

The data presented in this study are available on request from the corresponding author.

## Appendix A

**Table A1:** Supermarket outlets by size.

Small-Sized	Medium/Large-Sized
Small chain supermarkets, non-discount stores (Carrefour Express) Small chain supermarkets, discount stores (Día Discount Market)	Chain supermarkets and hypermarkets, non-discount stores (Carrefour market and hypermarket, Coto, Disco, Jumbo, Vea, and Walmart) Chain supermarkets and hypermarkets, discount stores (Día Maxi) Independent (non-chain) supermarkets, (includes supermarkets owned by Chinese independent grocers called “Chinese supermarkets”)

## Appendix B

Selection of foods and beverages to be included in indicators of relative availability of healthy and unhealthy foods in Buenos Aires.

The INFORMAS protocol to estimate Food Availability in Supermarkets recommends the use of an indicator of relative availability of foods and beverages [36,37]. In this study, the indicator was adapted to be used in Argentina by including other healthy items besides fruits and vegetables. To assess whether the selected foods and beverages were representative of the foods and beverages in supermarkets in Buenos Aires, we have conducted a sub-study. Briefly, we measured the shelf length assigned to all the food and beverages available in five supermarkets of different size and chain. Food and beverage items were classified into three groups according to the dietary guidelines for the Argentinian population: those recommended to be consumed (G1), those of which the consumption should be moderated (G2) and those of which consumption should be limited (G3) [13,14]. Results from the indicator adapted for Argentina were similar to the ratio between the G1 and G3 shelf length, taking into account all the available foods and beverages in the stores (mean difference of 0.05, ranging from 0.00 to 0.10).
